# RNA m6A methylation regulators in liver cancer

**DOI:** 10.1186/s12935-023-03197-x

**Published:** 2024-01-02

**Authors:** Qiaoping Xu, Ning Ren, Lanqi Ren, Yibei Yang, Junjie Pan, Hongkai Shang

**Affiliations:** 1 Department of Clinical Pharmacology, Key Laboratory of Clinical Cancer Pharmacology and Toxicology Research of Zhejiang Province, Affiliated Hangzhou First People’s Hospital, Cancer Center, Westlake University School of Medicine, Hangzhou, 310006 China; 2 Fourth Clinical Medical College of Zhejiang, Chinese Medical University, Hangzhou, 310051 Zhejiang China; 3 The Fourth School of Clinical Medicine, Zhejiang Chinese Medical University, Hangzhou, China; 4 Department of the Fourth Clinical Medical College, Zhejiang Chinese Medical University, Hangzhou, China; 5 Department of Gynecology, Hangzhou First People’s Hospital, Hangzhou, China; 6 Department of Gynecology, Westlake University School of Medicine, Hangzhou, China

## Abstract

Liver cancer is one of the most common cancers in the world and a primary cause of cancer-related death. In recent years, despite the great development of diagnostic methods and targeted therapies for liver cancer, the incidence and mortality of liver cancer are still on the rise. As a universal post-transcriptional modification, N6-methyladenosine (m6A) modification accomplishes a dynamic and reversible m6A modification process, which is executed by three types of regulators, methyltransferases (called writers), demethylases (called erasers) and m6A-binding proteins (called readers). Many studies have shown that m6A RNA methylation has an important impact on RNA metabolism, whereas its regulation exception is bound up with the occurrence of human malignant tumors. Aberrant methylation of m6A RNA and the expression of related regulatory factors may be of the essence in the pathogenesis and progression of liver cancer, yet the precise molecular mechanism remains unclear. In this paper, we review the current research situations of m6A methylation in liver cancer. Among the rest, we detail the mechanism by which methyltransferases, demethylases and m6A binding proteins regulate the occurrence and development of liver cancer by modifying mRNA. As well as the potential effect of m6A regulators in hepatocarcinogenesis and progression. New ideas and approaches will be given to the prevention and treatment of liver cancer through the following relevant research results.

## Introduction

Liver cancer is a common malignancy, ranking sixth in incidence and fourth in mortality among all cancers [1]. Hepatocellular carcinoma(HCC) and cholangiocarcinoma(CCA) are two major subtypes of primary liver cancer [2]. Among them, HCC accounts for 75–85% of primary liver cancer. Patients who are in the early-stage of liver cancer may be cured with surgical excision or liver transplantation. But in reality, most patients were not amenable to surgical resection, and worse still, the treatment they received had little effect. Despite recent advances in medical technology, dramatic improvements in medical level and improvements in cancer survival rates, liver cancer is still one of the few cancers in which the overall mortality rate is on the rise [3].

In recent years, continuous efforts have been made to map and quantify various RNA modifications within the transcriptome range, and RNA modification has been found to be a highly dynamic and reversible process that participates in important biological processes. Among more than 100 RNA modifications identified so far, m6A is one of the most common post-transcriptional modifications in eukaryotic RNA transcripts and long non-coding RNAs (lncRNAs), which plays a dominant role in the self-renewal, proliferation and spread of tumor cells [4,5]. Moreover, based on the deepening research of DNA methylation and histone modification analysis, researchers in the field of epigenetics have begun to pay attention to RNA modification represented by m6A [6].

Although RNA-based therapy exploration is still in its early stages, it has gained widespread recognition because methylated RNA molecules play an important role in regulating almost all aspects of cell biology and may be specifically recognized. This indicates that RNA-based modification therapy may be a valuable approach in the field of cancer treatment, and m6A has been recognized as a new mechanism for tumor related gene regulation. The tissue specific m6A spectrum of some transcripts and transcription sites may contain new cancer diagnostic and prognostic markers [7].

According to the latest progress, upregulation and downregulation of m6A methylation and its regulatory factor expression play an important role in the occurrence and development of liver cancer [8,9]. Therefore, this article discusses the relationship between m6A methylation modification, related regulators and liver cancer, in order to provide new approaches and methods for the prevention, early diagnosis and treatment of liver cancer.

## Biological classification and current status of liver cancer

The fifth edition of the World Health Organization (WHO) classifies HCCs into eight distinct histological subtypes: steatohepatitic, fibrolamellar, scirrhous, clear cell type, macrotrabecular massive, chromophobe, neutrophil-rich and lymphocyte-rich [10]. Each type has its own unique clinicopathological results [11,12].

However, the non targeted mutations in HCC increase the difficulty of clinical management [13]. Therefore, the most widely used HCC staging system in clinical practice is Barcelona clinical liver cancer (BCLC) staging system, which divides HCC into five stages: 0, A, B, C, D, and it clarifies the treatment standard of each tumor stage and the life expectancy of patients [14,15]. Studies have shown that the overall 5-year survival rate of HCC is decreasing. Although the survival rate of early-stage HCC is still high, the prognosis of advanced HCC is not optimistic [16]. And when it comes to prognosis, the major obstacle to improving the prognosis of patients with HCC is that metastasis and recurrence occur in 60 to 80% of patients [17]. In addition, studies have shown that HCC survival rates with high expression of HCC stem cell markers such as CD44, CD133, and CD90 are lower [17].

Currently recognized risk factors for HCC include chronic viral infections (such as hepatitis B virus and hepatitis C virus), exposure to toxins such as aflatoxin, alcoholic cirrhosis, and smoking [18–21]. There is also evidence that nonalcoholic fatty liver disease (NAFLD) is a relevant risk factor for HCC [18], as NAFLD is associated with obesity and metabolic syndrome (hypertension, type 2 diabetes). More importantly, it is usually accompanied by significant hepatic steatosis and inflammation, which undoubtedly promotes the progression of cirrhosis and ultimately leads to HCC [18,19].

Nowadays, the clinical treatment of liver cancer remains very challenging. Surgical resection is only suitable for 20 to 30% of patients with liver cancer, and tumor recurrence is common. When using molecular targeted therapy, the recommended drugs for patients with unresectable advanced HCC include sorafenib and lenvatinib, but they only prolong patient survival by 3 months [22]. Nivolumab is an anti-PD1 immune checkpoint therapy, which has been approved by FDA as a new second-line treatment for sorafenib-refractory HCC. It can boost the survival of HCC patients, but only 25% of them respond to the therapy [23].

According to the latest statistics, there are 841,000 new cases and 782,000 deaths of liver cancer each year [24]. Therefore, liver cancer is still a difficult disease to cure combined with late diagnosis and limited treatment options. Looking for high specificity and sensitivity of new markers facilitate in the early diagnosis of liver cancer, so as to improve the quality of life and the cure rate of patients is of great significance [25].Understanding the molecular mechanisms underlying the development of liver cancer is fundamental to accelerate future diagnostic and therapeutic inventions.

## Function of m6A and the role of three regulatory proteins

N6-methyladenosine (m6A) methylation is a kind of epigenetic modifications of RNA [26], it can adjust the RNA transcription, editing, translation and stability [27]. The distribution of m6A is found across more than 7000 mRNA and multiple non-coding RNA (ncRNA) transcripts in human cells [28]. In mRNA, m6A locates in the coding sequence (CDS) and 3' end of the translation Sect. (3' UTR) at most, especially near the termination codon. In addition, in the long non-coding RNAs (lncRNAs), microRNAs (miRNAs) and circular RNAs (circRNAs), we can also detect abundant m6A modification sites [26,29]. These modifications are subjected by time and space, and the fact that they are asymmetrically distributed and dynamically reversible in nature suggests that these proteins have great potential in regulating biological processes [30]. There are three kinds of proteins that adjust m6A modification: writers, erasers, and readers [31,32]. Writers are methyltransferases, including METTL3, METTL5, METTL14, WTAP, RBM15 and ZC3H13. Erasers are demethylases, including FTO and ALKBH5. Readers are m6A-specific methylation-reading proteins, including IGF2BP1/2/3, YTHDF1/2/3 and ELAVL1.

These three regulatory proteins play their respective roles in co regulating m6A modification. The main function of m6A writer is to catalyze the m6A modification of adenosine on mRNA. These proteins are not isolated, but rather form complexes to collectively perform catalytic functions. The main function of m6A eraser is to demethylate and modify bases that have undergone m6A modification. The m6A reader regulates the biological behavior of mRNA and performs corresponding functions by reading m6A methylation Research has shown that m6A disorder is strongly linked to the occurrence of malignant tumor [29], and these three types of dysregulation proteins are often seen in cancer [26–32]. They play an essential role in accelerating and/or restraining cancer, influencing cancer progression and patient prognosis by adjusting different downstream molecules and signaling pathways [31].

## m6A writers

Writer is a kind of m6A methyltransferase compound that is used to mount most mRNA m6A modifications [33]. METTL3 and METTL14 are its main kernel components, which play vital roles in different methyltransferase complexes. METTL3 (methyltransferase Like 3) is deemed to be the main enzyme that exerts methyltransferase activity in the protein complex. It binds to a second support enzyme, METTL14, to form a heterodimer that preferentially methylates the GGACU domain [33,34]. METTL14 has the function of stabilizing conformation and promoting RNA binding. In addition to the constitutive methyltransferase domain, METTL3-METTL14 also carries a C-terminal arginine-glycine repeat (RGG), the deletion of which reduces the catalytic activity of METTL3-METTL14 [35–37]. WTAP, the third component of writer, acts on the junction of METTL3 and METTL14, contributing to anchor the methyltransferase complex in the nuclear spot (also known as splicing factor compartment) and promote m6A deposition [33–37]. Subsequently, new writers, such as RBM15(B), HAKAI, METTL16, and KIAA1429 (VIRMA) have been identified [35]. RBM15 (or RBM15B) recruits m6A compounds by combining with the U-rich region, and may facilitate the development of specific RNA methylation [34,35,38]. Whereas viral-like m6A methyltransferase association (VIRMA/KIAA1429) plays an important role in guiding the heterodimer to its target region. Recently, VIRMA is verified to preferentially mediate m6A deposition at the 3’UTR near the stop codon, which is partly related to alternative polyadenylation via [33,38]. Methyltransferase-like protein 16 (METTL16), a newly discovered RNA methyltransferase, can catalyze the installation of m6A on the 3’UTR and A43 of the U6 small nuclear RNA mRNA [38,39]. HAKAI is a novel methyltransferase which interacts with WTAP and is indispensable for m6A methylation [35]. Knock-down of HAKAI in HeLa cells down-regulated the level of m6A [39]. ZC3H13 and its cognate protein FLacc are also involved in m6A mounting by promoting WTAP localization and m6A deposition [35]. The Table 1 summarizes the functions of m6A writers in liver cancer.

**Table 1:** Functions of m6A ‘writers’ in liver cancer

Regulator	Effect on m6A modification	Expression change	(Refs.)
METTL3	Catalytic core of methyltransferase	Up	36, 37
METTL14	Forms a heterodimer with METTL3 without catalytic action, enhancing the activity of METTL3	Down	38–40
WTAP	Anchors the methyltransferase complex in the nuclear speckles	Up	^36–40^
RBM15/15B	Binding to the U-rich region to recruit m6A complexes	Up	^37,38,41^
KIAA1429(VIRMA)	Interacts with WTAP and recruits m6A to the 3’UTR	Up	36,41
METTL16	Catalyzes m6A installation in the 3’UTR in mRNA and on A43 of U6 small nuclear RNA	Up	^41,42^
HAKAI	Necessary for the methylation of m6A	Up	^42^
ZC3H13	Promotes the WTAP localization and m6A deposition	Down	^38^

m6A, N6-methyladenosine; METTL3, methyltransferase-like protein3; METTL14, methyltransferase

-like protein 14; WTAP, Wilms tumor 1-associated protein; RBM15/15B, RNA binding motif protein 15/15B; KIAA1429 also called VIRMA, vir-like m6A methyltransferase-associated protein; METTL16, methyltransferase-like protein16;HAKAI, Casitas B lineage lymphoma transformation sequence-like protein 1 (CBLL1); ZC3H13, zinc finger CCCH domain-containing protein 13

## m6A erasers

As m6A demethylases (called erasers), FTO and ALKHB5 play oncogenic and inhibitory roles in tumorigenesis, respectively [40,41]. FTO belongs to the α-ketoglutarate dependent hydroxylase superfamily and is the first demethylated enzyme to be discovered, which catalyzes not only the demethylation of thymidine and uracil bases in DNA, but also the modification of N6, 2’o-dimethyladenosine (m6Am) and m6A in snRNAs and mRNAs [42,43]. These demethylation functions indicate the dynamic and reversible nature of m6A modification [44]. The FTO protein is predominantly present in the nucleus, partially co-localized with nuclear spots. In addition, the presence of FTO is also found in the cytoplasm of several different cell types, suggesting a potential role for FTO in regulating cytoplasmic mRNA expression [40]. In terms of the expression of FTO, its down-regulation significantly advance the content of m6A in total RNA, and the increased expression may restrain the growth and metastasis of liver cancer cells [42,45]. Furthermore, it has been shown that FTO adjusts liver adipogenesis through FTO-dependent m6A demethylation in FASN [45].

AS a member of the AlkB family of non-heme Fe(II)/α-KG-dependent dioxygenases, ALKBH5 mainly locates on nuclear speckles. ALKBH5-mediated m6A demethylation adjusts gene expression through multiple events influencing RNA metabolism such as pre-mRNA processing, mRNA decay and translation [46, 47]. Studies have shown that when the expression level of ALKBH5 is reduced in liver cancer, it plays a role in inhibiting malignant tumors, while when the expression level is significantly increased, it indicates poor prognosis of liver cancer [48,49]. In summary, the abnormal expression of FTO and ALKBH5 affects the expression level of m6A, which further affects the development of cancer, indicating that they have the potential to be prognostic biomarkers for various cancers. The functions of m6A erasers in liver cancer are listed in Table 2**.**

**Table 2:** Functions of m6A ‘erasers’ in liver cancer

Regulator	Effect on m6A modification	Expression change	(Refs.)
FTO	Removes m6A and m6Am modification, regulates pre-mRNA alternative splicing	Up/down	[45, 46]
ALKBH5	Removes m6A modification, regulate mRNA processing, metabolism and export	Up/down	[49, 50]

m6A, N6-methyladenosine; FTO, fat mass and obesity-associated protein; ALKBH5, AlkB homolog 5

## m6A readers

“Readers” refer to proteins capable of identifying and combining m6A modification, and it can mediate the regulation of m6A modification in gene expression mainly by influencing the fortune of targeted RNAs. In brief, reader proteins are of great importance in performing m6A functions [50]. The reading proteins of m6A are primarily YTH domain-containing proteins. There are three major classes of proteins that contain the YTH domain: YTHDF family proteins, YTHDC1 and YTHDC2 proteins [51,52]. YTHDF family proteins and YTHDC2 are diffusely distributed in the cytoplasm, while YTHDC1 is plentiful in nucleus. YTH proteins have the function of raising translation efficiency and furthering mRNA degradation [53,54].

YTHDF1 can combine with specific recognition in 3 'UTR of m6A, and recruit 43S pre-initiation compound to initiate the translation process. Furthermore, it is found that YTHDF1 can facilitate ribosome loading of targeted mRNA, indicating that YTHDF1 can enhance mRNA translation [50,55]. In heat shock-induced transcripts, nuclear YTHDF2 maintains 5’UTR methylation by restricting demethylation of the m6A demethylase FTO [53,56]. YTHDF3 also plays an essential role, helping to regulate the fate of mRNAs by manipulating YTHDF1 and YTHDF2. Besides, YTHDF3 interacts with the 40S and 60S ribosomal subunits by cooperating with YTHDF1, thereby facilitating the translation of methylated mRNAs [53,57]. In mammals, YTHDC1 is a recruiter of mRNA splicing factors, which can competitively recruit two splicing effectors: the serine/arginine-rich splicing factors 3 and 10 (SRSF3 and SRSF10) [51]. Unlike YTHDF2, YTHDC2 increases the local concentration of the RNA decay machinery, thereby to regulate RNA stability in an RNA-independent manner [50].

In addition to the YTH domain family, hnRNPC, hnRNPG and hnRNPA2B1 members of the heterogeneous nuclear ribonucleoprotein family are identified as m6A readers that regulate alternative splicing events [35]. HnRNPC protein is a nuclear RNA binding protein involved in the processing of pre-mRNA, whereas HnRNPG protein can selectively combine with m6A modified RNA [58]. HNRNPA2B1 enhances METTL3-dependent microRNA processing and also participates in the process of interaction with the microprocessor mechanism [59,60]. IGF2BPs function to enhance the mRNA stability and mediate translation in an m6A-dependent manner [61,62]. The functions of m6A readers in liver cancer are listed in Table 3.

**Table 3:** Functions of m6A ‘readers’ in liver cancer

Regulator	Effect on m6A modification	Expression change	(Refs.)
YTH domain family			
YTHDF1	Enhances m6A-modified RNA translation	Up	^53,58^
YTHDF2	Promotes m6A-modified RNA degradation	Up	^56,59^
YTHDF3	Accelerates m6A-modified RNA translation and degradation	Up	^56,60^
YTHDC1	Regulates m6A-modified RNA splicing and export	Up	^54^
YTHDC2	Promotes m6A-modified RNA translation efficiency	Up	^53^
HNRNP family			
HNRNPC and HNRNPG	Regulates the abundance and alternative splicing of target genes	Up	^61^
HNRNPA2B1	Promotes the processing of primary miRNA	Up	^62,63^
IGF2BP1-3	Stabilizes m6A-modified mRNA	Up	^64,65^

m6A, N6-methyladenosine; miRNA, microRNA; YTH, YT521-B homology; YTHDF1, YTH domain family 1; YTHDC1, YTH domain containing 1; HNRNP, heterogeneous nuclear ribonucleoprotein protein; IGF2BP, insulin-like growth factor 2 mRNA binding protein

## Research progress on the relationship between various m6A regulators and liver cancer

In recent years, researchers have found that m6A RNA modification, as an epigenetic regulator, participates in the occurrence of tumors, including liver cancer, breast cancer and gastric cancer, which dynamically and reversibly control the structure and function of RNA [63]. The m6A adjusts tumor proliferation, metastasis and other processes.

The results show that both m6A levels and the methylase METTL3 are significantly elevated in lung adenocarcinoma patients and lung cancer cells [64]. In bladder cancer, mettl3 promotes bladder tumor angiogenesis via modulating TEK and VEGF-A [65]. The expression of FTO is up-regulated in gastric cancer tissues, which may be associated with the metastasis and progression of gastric cancer [66]. ALKBH5-modified HMGB1-STING activation is involved in the development of radiation-induced liver disease through innate immune response. And ALKBH5-mediated LINC02551 m6A methylation is necessary for HCC growth and metastasis [67,68]. According to reports, YTHDF1 enhances the growth of HCC cells by activating the PI3K/AKT/mTOR signaling pathway. YTHDF1 is highly expressed in HCC and correlated with HCC grade. Depletion of YTHDF1 significantly inhibited the proliferation, migration, invasion, and cell cycle progression of HCC cells [69]. In addition, YTHDF3 exerts anti-liver fibrosis effect by up-regulating the expression of PRDX3 [70]**.**

The expression of m6A regulatory factors may be associated with the malignant degree and poor prognosis of liver cancer. Unfortunately, to date, there are no specific inhibitors targeting m6A regulatory proteins other than FTO. Further research is needed to develop large-scale structural chemical screening of specific targeted deregulated m6A regulatory protein inhibitors. Anyway, m6A regulatory factors may be potential molecular therapeutic targets for liver cancer. Therefore, we summarize the major roles of some important m6A regulators in the liver cancer.

**Fig. 1:**
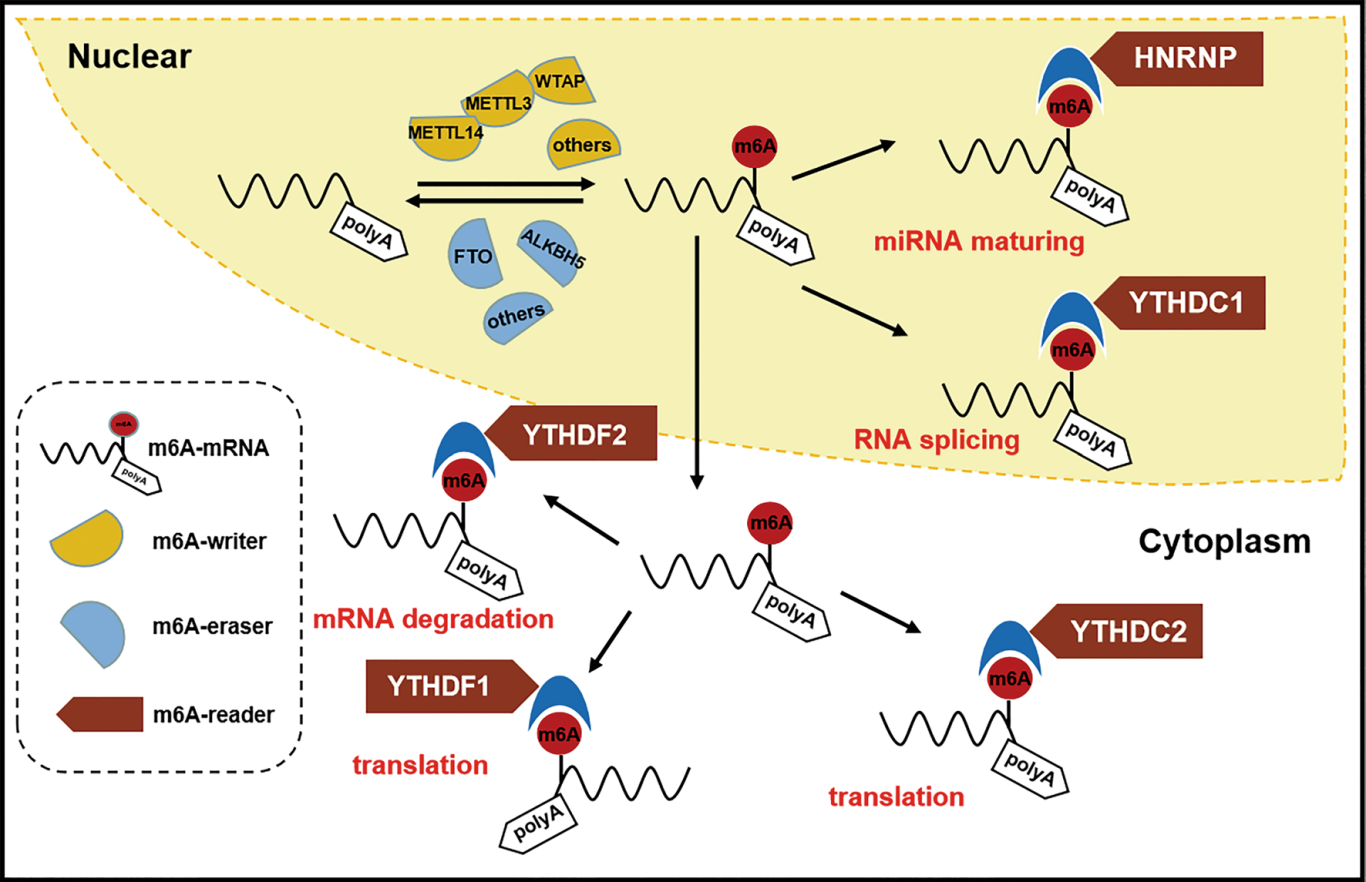
M6A dynamic regulation. Simple model of m6A Dynamic regulation. m6A methylation is regulated by methyltransferase (“Writers”), demethylases (“Erasers”) and m6A-binding proteins (“Readers”). Methyltransferase METTL3/14, WTAP, VIRMA, ZC3H13, RBM15/15B etc. mainly catalyze the modification of mRNA by m6A. Demethylases, including FTO and ALKBH5, are used to demethylate bases modified by m6A. The main function of m6A-binding proteins is to recognize sites modified by m6A, thereby activating downstream regulatory pathways such as RNA degradation and miRNA processing. The m6A site is bound to different readers to accommodate different functions

The following is a simplified model of M6A dynamic regulation (Fig. 1).

## METTL3 in liver cancer

It has been suggested that METTL3 plays a vital role in different stages of m6A RNA life cycle and is associated with tumorigenesis [71–73]. METTL3 expression is up-regulated in a variety of human HCC cell lines, leading to increased mRNA m6A modification and promoting HCC progression [73,74]. The expression of METTL3 increased gradually from grade 1 to grade 3, but the difference between grade 3 and grade 4 was not statistically significant. More importantly, METTL3 expression was not associated with HBV or HCV viral infection, suggesting that METTL3 upregulation may be a universal feature in the development of HCC with different etiologies [74] Overexpression of METTL3 can promote the proliferation and migration of HCC mainly because METTL3 can regulate the m6A modification of SOCS2 mRNA, it promotes the occurrence and development of liver cancer by reducing the stability of SOCS2 mRNA through m6A-YTHDF2-dependent pathways [75,76].

Mechanistically, we find that METTL3-mediated m6A modification of abnormal spindle-microcephaly (ASPM) mRNA promoted its expression in hepatocellular carcinoma (LIHC). Silencing METTL3 can inhibit the proliferation, migration, and invasion of LIHC cells [77].

In HCC, METTL3 is an oncogene, while METTL14 is a tumor suppressor gene. The overall survival (OS) time, recurrence-free survival (RFS) time, progression-free survival (PFS) time and disease-specific survival (DSS) time of HCC patients with low METTL14 expression are shorter than those of patients with high METTL14 expression [78]. This demonstrates the prognostic value of METTL14 in HCC.

In summary, as the main catalytic enzyme of m6A methylation, METTL3 has a complex mechanism involving multiple signal transduction pathways and multiple molecular expression. At present, the relationship between Mettl3 and Mettl14 and liver cancer and the exact mechanism still need to be further studied.

## WTAP in liver cancer

WTAP is a conserved nuclear protein that acts as a chaperone in type 1 Wilms tumor. WTAP is able to stabilize METTL3 and METTL14 and localize them in nuclear speckles [79]. We can often see WTAP disordered in cancer with cancer specific pattern. For example, WTAP is up-regulated in stomach adenocarcinoma (STAD), kidney renal clear cell carcinoma (KIRC) and hepatocellular carcinoma (HCC), while down-regulated in uterine corpus endometrial carcinoma (UCEC), thyroid cancer (THCA), lung adenocarcinoma (LUAD), and bladder urothelial carcinoma (BLCA) [72,80]. WTAP is also an oncogene associated with heat shock protein 90 in AML and diffuse large B-cell lymphoma [81].

However, Wilms tumor 1 associated protein (WTAP), an important component of m6A methylation, is understudied in HCC. Research shows that the expression levels of WTAP in liver cancer tissues are significantly higher than that in the adjacent normal tissues, which is significantly connected with the clinical stage in patients with HCC [79,80,82,83]. The high expression of WTAP in HCC also reveals poor prognosis and patient survival [84]. Mechanically, WTAP-guided m6A modification regulates the G2/M phase of HCC cells through the HuR-ETS1-p21/p27 axis, thereby accelerating the occurrence of HCC [79]. The overexpression of WTAP partially reversed the inhibitory effect of miR-139-5p on the growth and invasion of HCC cells [83]. Therefore, it is reasonable to believe that WTAP could be a potential therapeutic target for HCC treatment.

**Fig. 2:**
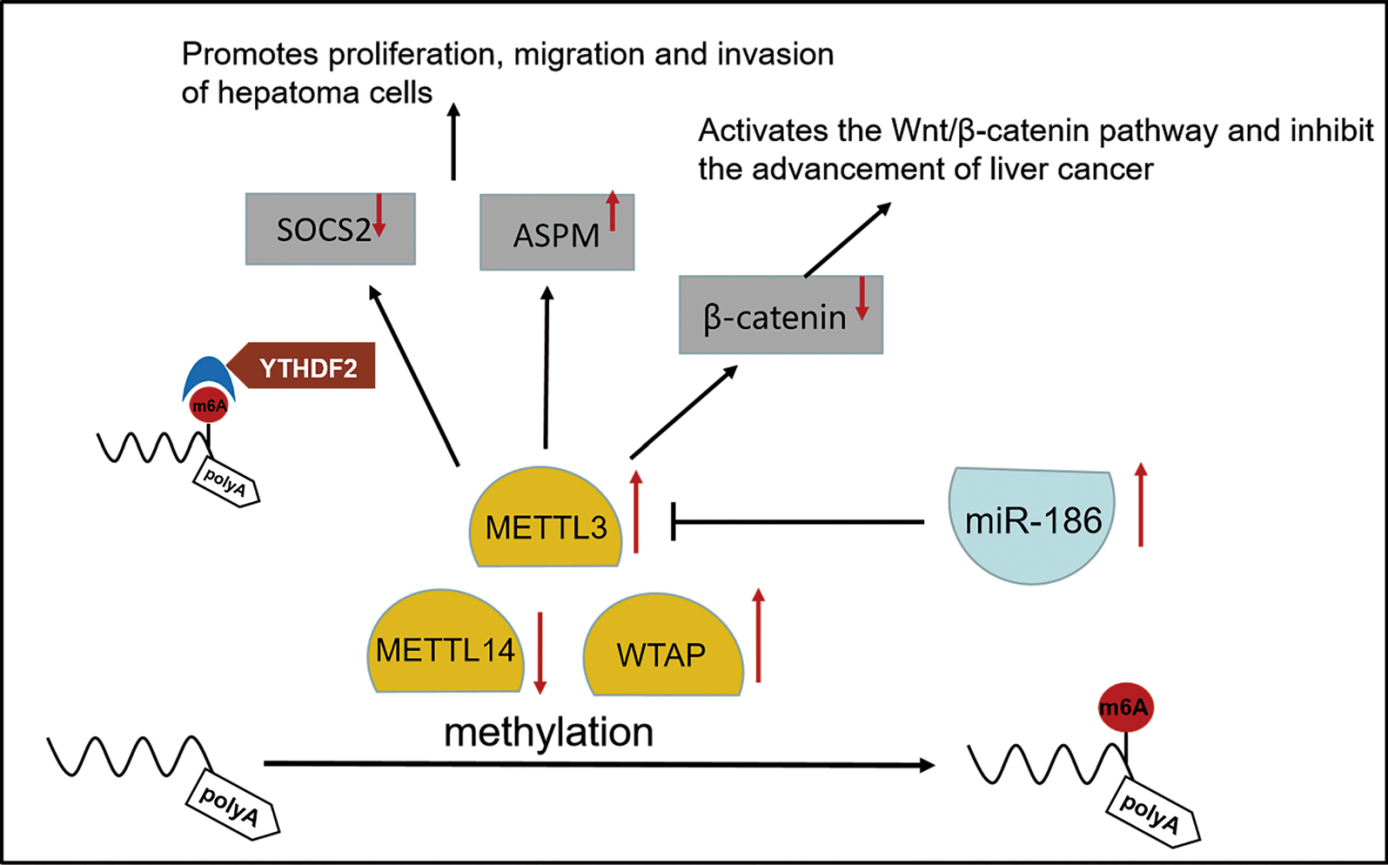
primary contributions of m6A methylases in liver cancer. Major roles of m6A methylases(METTL3、METTL14、WTAP) in the occurrence and development of liver cancer. Increased expression of METTL3: **A** reduces the stability of SOCS2 mRNA through m6A YTHDF2-dependent pathway, thus promoting the occurrence and development of liver cancer; **B** promotes the modification of ASPM mRNA and promote the proliferation, migration and invasion of hepatoma cells. Moreover, overexpression of miR-186 significantly inhibited the expression of METTL3, thereby affecting the expression of Wnt/β-catenin pathway-related proteins such as β-catenin, thus promoting the proliferation, migration and invasion of hepatoma cells. METTL14 is a tumor suppressor gene, and its decreased expression may promote hepatocarcinogenesis. The up-regulated expression of WTAP in HCC also indicates poor prognosis and poor patient survival

The following is a summary of the primary contributions of m6A methylases in the occurrence and progression of liver cancer (Fig. 2).

## FTO in liver cancer

The alternative splicing function of FTO plays a role in mRNA processing including RNA modification, transcriptome regulation and translation. Although the role of the FTO gene in the tumor research is still in its early stages, a growing body of evidence suggests that FTO associated with the occurrence and development of a wide variety of tumor and prognosis [41,85]. As an m6A demethylase, FTO has a carcinogenic effect in a variety of human malignant tumors such as lung cancer and colon cancer, and enhances the proliferation and invasion of cancer cells [86].

The existing studies have not clarified the specific mechanism of FTO in the occurrence and development of liver cancer, and many research results point to the dual role of FTO in the occurrence and development of liver cancer. A study by YE et al. found that FTO accelerated the proliferation and mobility of human hepatoma cell line HepG2 in vitro. It suggests that it may promote the proliferation and migration of hepatoma cells. In addition, upregulation of FTO expression in patients with liver cancer is associated with high Edmondson grade, which is an independent prognostic factor for liver cancer [87]. The study by Zhao et al. showed that patients with decreased FTO expression had shorter overall survival and tumor-free survival compared with patients with normal FTO expression [88]. Melanie J. Mittenbühler et al. tended to study the function of liver FTO to play a protective role in the development of HCC in vivo. The results indicate that FTO deficiency affects not only HCC development (increased number of tumors) but also HCC progression (increased number of large tumors) [43]. In addition, FTO can also inhibit HCC tumorigenesis and metastasis through circGPR137B/miR-4739/FTO feedback loop [89].

To sum up, the above studies suggest that FTO may be an important prognostic factor for HCC patients and may be a new biomarker of HCC. However, in order to better understand the relationship between liver cancer development and FTO, further research is needed.

## ALKBH5 in liver cancer

ALKBH5, another RNA demethylase, plays a role in many cancers by regulating a variety of biological processes, such as proliferation, migration, invasion, metastasis and tumor growth [90]. However, the role of ALKBH5 in human cancer is controversial. The expression is up-regulated or down-regulated in different types of cancer, playing a carcinogenic or anticancer role [90–95]. In lung cancer, patients with high expression of ALKBH5 have shorter overall survival (OS) than those with low expression of ALKBH5. This suggests that ALKBH5 is upregulated in lung cancer and is associated with poor prognosis in lung cancer patients. Down-regulation of ALKBH5 inhibits the angiogenesis of lung cancer cells in vitro and in vivo [94]. In breast cancer and glioblastoma, ALKBH5 plays a carcinogenic role [93,95]. Whereas in pancreatic cancer, ALKBH5 acts as a tumor suppressor, and its up-regulation can improve the survival rate of pancreatic cancer patients [91]. Recent studies have also shown that ALKBH5 is a novel tumor suppressor capable of inhibiting colon cancer invasion and metastasis [95]. To sum up, ALKBH5 plays different roles in different tumor types, and its mechanism of action is complex.

**Fig. 3:**
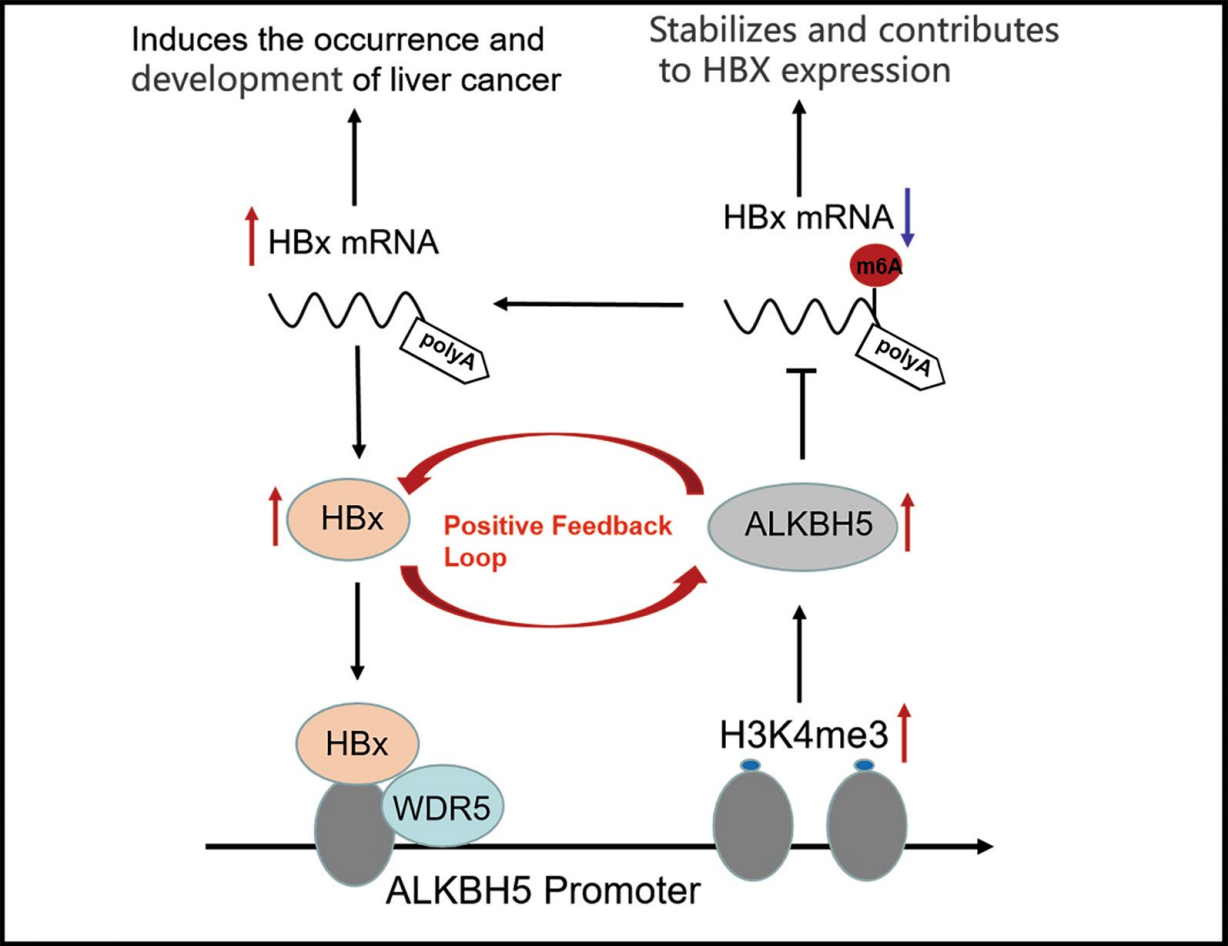
A positive correlation between HBx and ALKBH5 in HBV-HCC tissues. HBx-ALKBH5 may form a positive feedback loop and participate in HBV-induced hepatocarcinogenesis. HBV upregulates the expression of ALKBH5 through the HBx-WDR5-H3K4me3 axis, and ALKBH5 promotes the stabilization of HBx mRNA by reducing m6A fluctuations, thus forming a positive feedback loop

Studies have shown that ALKBH5 expression is decreased in HCC and is an independent prognostic factor for poor survival in HCC patients [96]. In terms of function, ALKBH5 can inhibit the proliferation and invasion of HCC cells in vitro and in vivo. Mechanistically, ALKBH5 acts as a tumor suppressor mediating m6A demethylation, leading to post-transcriptional repression of LY6/PLAUR domain containing protein 1 (LYPD1), which is recognized and stabilized by the m6A effector IGF2BP1. Overall, dysregulation of ALKBH5/LYPD1 axis promotes HCC progression [96]. Moreover, Qu et al. found that HBx-ALKBH5 may form a positive-feedback loop to participate in the HBV-induced hepatocarcinogenesis [48]. From the perspective of mechanism, HBV infection induces high expression of ALKBH5 through H3K4me3 modification of ALKBH5 gene promoter mediated by HBx in a WDR5-dependent manner. Increased ALKBH5 protein catalyzes the m6A demethylation of HBx mRNA, thereby stabilizing and accelerating the high expression level of HBx. In addition, there is a positive correlation between HBx and ALKBH5 in HBV-HCC tissues, and ALKBH5 knockdown significantly inhibits HBV-driven tumor cell growth and migration in vitro and in vivo [48]. The possible mechanism is presented in Fig. 3.

## YTHDF2 in liver cancer

YTH domain family proteins are collected by m6A and are associated with several mRNA metabolism, including mRNA splicing, translation and mRNA degradation. Therefore, YTH domain family proteins may be involved in many tumor physiological processes [97]. YTHDF2 is the first characterized m6A reader that accelerates the decay of m6A-modified transcription scripts by facilitating direct recruitment of the CCR4-NOT complex [57,98]. Many studies have indicated that YTHDF2 may have an intrinsic role in HCC genesis and affect the long-term outcome after HCC resection, for example, by causing sporadic recurrence [97,99–101].

In HCC, the expression levels of YTHDF1, YTHDF2 and YTHDF3 proteins increase with the progression of HCC stage, suggesting that these three proteins may participate in the disease progression of HCC patients [97]. Notably, both mRNA and protein expression of YTHDF2 is significantly higher in HCC tissues than in non-cancerous tissues. In addition, high expression of YTHDF2 in HCC correlates with clinical factors of poor prognosis (high clinical stage, histological grade, and T grade) and negatively correlates with OS and recurrence-free survival (RFS) after curative surgery [100,102].

The research of Hou et al. shows that YTHDF2 silencing in human HCC cells or ablation in mouse hepatocytes caused inflammation, vascular reconstruction and metastasis. In mechanism, YTHDF2 handles the decay of m6A-containing interleukin 11 (IL11) and serine protease inhibitor family E member 2 (SERPINE2) mRNAs, which are responsible for the destruction and vascular normalization of inflammation-mediated malignancies [99]. Hypoxia is a common feature of many solid cancers, including HCC. Hypoxia inducible factor (HIF) can adjust self-renewal of cancer stem cells, tumor progression and chemotherapy resistance. YTHDF2 transcription is subordinate to hypoxia-inducible factor-2α (HIF-2α). Studies have shown that YTHDF2 is the only mRNA whose expression level is significantly reduced in HCC cells under hypoxic conditions. And YTHDF2 may negatively regulate the stability of EGFR mRNA by binding to the m6A site in the 3’UTR of EGFR mRNA, thereby impairing the MEK/ERK pathway and hindering cell proliferation and growth [98]. Administration of a HIF-2α antagonist (PT2385) can restore the epigenetic mechanism of YTHDF2 programming and inhibit liver cancer [99].

In conclusion, YTHDF2 may be an important biomarker for the diagnosis and prognosis of liver cancer. However, the specific role of YTHDF2 in liver cancer needs further study.

## IGF2BP1 in liver cancer

Insulin-like growth factor 2 (IGF2) mRNA-binding protein 1 (IGF2BP1) is a primary member of the conserved IGF2BP RNA-binding family proteins. IGF2BP1 is rarely observed in adult life but is upregulated or regenerated in cancer [103]. Recent studies have shown that IGF2BP1 dependent mRNA encode oncoproteins that are essential for neoplastic transformation and cancer cell progression. IGF2BP1 influences the proliferation and tumorigenicity of leukemia cells through the crucial self-renewal regulators HOXB4 and MYB and the aldehyde dehydrogenase ALDH1A1 [104]. IGF2BP1 can promote melanoma metastasis mediated by extracellular vesicles. [105]. Meanwhile, IGF2BP1 promotes the growth of tumor cells such as liver cancer by enhancing the expression of a variety of serum response factor (SRF) target genes, including PDLIM7, FOXK1, MKI67 and MYC [106]**.** IGF2BP1 is also important for mRNA stability and translation of several other oncogenes, including glioma related oncogene homologue 1 (GLI1), Myc and CD445 [107].

It has been reported that IGF2BP1 expression is significantly higher in HCC than in adjacent benign tissues [108]. Consistent with this, overexpression of IGF2BP1 in human HCC is associated with poor survival of HCC patients and positively correlated with tumor T and N grades [103,107,109]. From the perspective of mechanism, IGF2BP1 physically interacts with the corresponding mRNA, leading to its stabilization, increased expression, and eventually exhibiting a cancerous phenotype [109]. In addition, we found that GLI1 is a target of IGF2BP1 in HCC cells. As a transcriptional activator, GLI1 is of great importance for the development of a variety of malignant tumors. Genes downstream of GLI1 have been found to be involved in cell growth and invasion. And it should be noted that GLI1 mRNA and IGF2BP1 bind to LINC01093 in the same region. LINC01093 impedes GLI1 mRNA from binding to IGF2BP1 by competitive binding to IGF2BP1, thereby inhibiting the expression of GLI1 downstream genes and playing a crucial role in restraining tumor proliferation and metastasis [110]. Therefore, LINC01093-IGF2BP1-GLI1 axis provides a potential target for the treatment of HCC in the future.

**Fig. 4:**
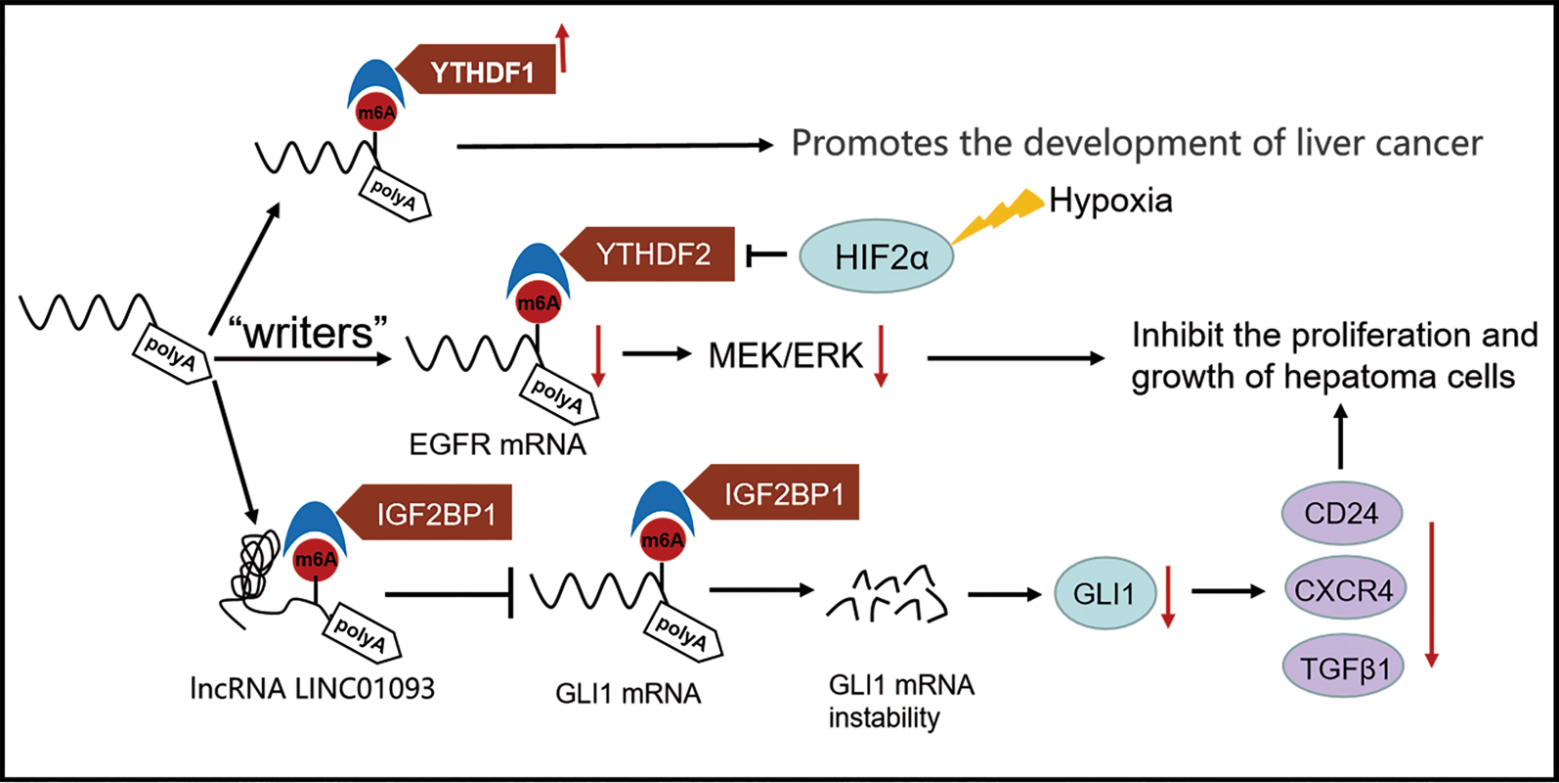
Primary contributions of m6A methylases in liver cancer

The following is a summary of the primary contributions of m6A methylases in the occurrence and progression of liver cancer (Fig. 4).

## Other m6A regulators in liver cancer

The expression of m6A RNA regulatory factor is closely associated with the malignant clinical features of HCC, and its gene changes often indicate poor clinical prognosis of HCC patients [111–113]. Therefore, the expression level of m6A RNA methylation gene may become a new biomarker for predicting the prognosis of liver cancer, which is of great significance in tumor research. Apart from the above-mentioned mainstream regulatory factors, the following regulatory factors can also affect the occurrence and development of liver cancer.

Wu et al. found that ZC3H13 may be a potential tumor suppressor gene of HCC. ZC3H13 lower expression in HCC associated with the poor prognosis of HCC. In addition, ZC3H13 level is significantly and positively correlated with tumor immune cell infiltration, immune cell biomarkers and immune checkpoint expression [114]. In general, the N6-methyladenosine RNA methylation regulator ZC3H13 may serve as a novel biomarker and therapeutic target for HCC.

Su et al. found that METTL16 plays a vital oncogenic role in HCC and that overexpression of METTL16 is associated with poor prognosis. METTL16 gene deletion significantly inhibited the growth, migration and invasion of human HCC cells, and significantly inhibited tumor growth in vivo, accompanied by a significant reduction in global m6A and translation [115]. Meanwhile, eIF3a/3b, as a METTL16 target, also plays a potential carcinogenic role in HCC. Therefore, targeting METTL16/eIF3a/eIF3b axis provides a new therapeutic strategy for the treatment of liver cancer. In addition, KIAA1429 also inhibits ID2 by up-regulating the m6A modification of ID2 mRNA, thus facilitating the migration and invasion of liver cancer [116]. However, the exact mechanism of these regulatory factors remains to be further studied.

## Prospects and conclusions

m6A methylation directly or indirectly affects cell proliferation, metastasis, invasion and apoptosis, and is involved in the pathogenesis of many diseases, especially cancer. There are three types of regulators that perform dynamically reversible m6A modification processes: methyltransferases (known as writers), demethylases (known as erasers), and m6A-binding proteins (known as writers). During the development of liver cancer, they play a crucial part in regulating RNA transcription, splicing, processing, translation and decay. Accumulating evidence indicates that m6A regulators is of great significance in liver cancer. Clearly, different types of liver cancer have different molecular features and different prognosis. Therefore, it is reasonable to speculate whether there are differences in m6A RNA methylation levels, regulatory factor expressions and related mechanisms among different molecular subtypes of liver cancer.

Although the m6A modification was initially confirmed in 1970, its functionality was not thoroughly studied until 2012. To this day, research on m6A in liver cancer is still in its early stages. The exact mechanism by which many upstream and downstream m6A regulatory factors regulate liver cancer is not fully understood, apart from the known regulatory effects of m6A methylation. Therefore, this article comprehensively reviews the currently popular m6A regulatory factors, analyzes their main roles in the occurrence and development of liver cancer, and reveals the expression changes of related regulatory factors, such as the significant upregulation of METTL3 expression, which is related to the poor prognosis of liver cancer, in order to provide new methods and pathways for the diagnosis and prognosis of liver cancer.

The m6A methylation regulatory factor can serve as a molecular target for liver cancer treatment. Taking YTHDF1 as an example, studies have found that knocking out YTHDF1 can significantly inhibit the proliferation, migration, and invasion of HCC cells, and enhance in vitro cell apoptosis based on liver cancer cases in TCGA. And silencing YTHDF1 can inhibit the growth of xenograft tumors in vivo. Research on its mechanism suggests that YTHDF1 may promote epithelial mesenchymal transition (EMT) and activate AKT/glycogen synthase kinase (GSK) -3 β/β- Chain protein signal transduction promotes invasive phenotype. Moreover, YTHDF1 plays an important role in the tumor microenvironment (TME), and YTHDF1 knockdown increases antigen-specific CD8 + T cell anti-tumor effects. So it proves that YTHDF1 can serve as a potential molecular target for liver cancer treatment.

In addition, a series of small molecule inhibitors targeting m6A regulatory factors (such as FTO, ALKBH5, METTL3, etc.) have emerged in recent years, with FTO being the most attractive target. This shows considerable hope for preventing cancer growth. Between 2012 and 2019, researchers developed and identified a series of FTO inhibitors, such as rhein, MO-I-500, methylclofenac (MA), fluorescein, 2-hydroxyglutaric acid (R-2HG), FB23, and FB23-2, which showed significant anti-tumor effects in vitro and in vivo. Since 2020, FTO inhibitors have been continuously upgraded and optimized, including CS1/CS2 and Dac51. They not only inhibit cancer cell proliferation and self-renewal of cancer stem cells, but also enhance anti-tumor immunity. Therefore, we have reason to believe that inhibitors targeting m6A regulatory factors may have enormous potential in the treatment and prevention of liver cancer.

The future challenge is to further enhance understanding of the complex networks related to liver cancer, continue to explore the molecular details of the function and potential molecular mechanism system of m6A regulatory factors in liver cancer, and how these details can improve the prospects of liver cancer treatment.

## Data Availability

Not applicable.
